# High-throughput identification of genes influencing the competitive ability to obtain nutrients and performance of biocontrol in *Pseudomonas putida* JBC17

**DOI:** 10.1038/s41598-022-04858-z

**Published:** 2022-01-18

**Authors:** Swarnalee Dutta, Yong Hoon Lee

**Affiliations:** 1 Division of Biotechnology, Jeonbuk National University, 79 Gobong-ro, Iksan-si, Jeollabuk-do 54596 Republic of Korea; 2 Advanced Institute of Environment and Bioscience, Plant Medical Research Center, and Institute of Bio-Industry, Jeonbuk National University, Jeonju-si, Republic of Korea

**Keywords:** Biotechnology, Microbiology

## Abstract

Elucidating underlying mechanisms of biocontrol agents (BCAs) could aid in selecting potent BCAs and increasing their biocontrol efficacy. Nutrient competition is an important biocontrol mechanism; however, essential nutrient sources, and contributing genes for nutrient competition still remain to be explored. *Pseudomonas putida* JBC17 (JBC17WT) suppressed green mold in satsuma mandarins by inhibiting conidial germination of *Penicillium digitatum* via nutrient competition. To analyze genes essential for biocontrol performance of JBC17WT, we generated a transposon (Tn)-mediated mutant library and selected mutants with the ability to suppress conidial germination. Several mutants in the genes of flagella-formation, including *fliR*, *fliH*, and *flgG*, increased biocontrol performance and enhanced inhibition of conidial germination. They lost swimming motility, exhibited increased growth and rapid carbon and nitrogen utilization than the wild type under nutrient-poor conditions. The nutrient competition assay using polytetrafluoroethylene cylinders revealed that conidial germination was inhibited by nutrient absorption under nutrient-poor conditions. In addition, genes, including amidohydrolase (*ytcJ*), tonB-dependent receptor (*cirA*), argininosuccinate synthase (*argG*), D-3-phosphoglycerate dehydrogenase (*serA*), and chaperone protein (*dnaJ*), were involved in the inhibition of conidial germination. The results of this study indicate that rapid and continuous absorption of nutrients by JBC17WT restrict nutrient availability for conidial germination on nutrient-limited fruit surfaces, thereby decreasing the chances of fungal spores infecting fruits. The high-throughput analysis of Tn mutants of this study highlighted the importance of nutrient competition and the genes that influence biocontrol ability, which contributes to the development of biocontrol applications.

## Introduction

Microorganisms that are used for biocontrol suppress diseases by direct interaction with pathogens through antibiotic compounds, siderophores, and lytic enzymes, and indirect mechanisms through induced systemic resistance^1–5^. Additionally, competition between biocontrol agents (BCAs) and pathogens for nutrients and niches is known to be an important mode of action, specifically in postharvest disease control of vegetables and fruits^6,7^.

To enter plant tissues, necrotrophic plant pathogens initially kill the host tissues and then colonize them by utilizing leaked nutrients. During the attachment and colonization stages of pathogens, non-pathogenic microorganisms can be potential competitors for nutrients and space^3,7,8^. Limiting carbon sources at the entry site reduced spore germination of fungal pathogens, leading to compromised survival and proliferation^9^. Nutrient competition for carbohydrates and nitrogen was essential for the biocontrol activity of yeasts, such as *Aureobasidium pullulans*, against postharvest diseases^10–12^. Iron-chelating siderophores produced by pseudomonads scavenged iron, thereby limiting the pathogen growth^13,14^. Although several studies have indicated nutrient competition mechanisms for the suppression of pathogens^15^, the intrinsic mechanism and nutrient sources crucial in the competition remain to be elucidated.

In our previous studies, *Pantoea agglomerans* 59–4 and *Pseudomonas putida* JBC17 (JBC17WT) suppressed postharvest blue mold and green mold diseases of *Citrus unshiu* (Satsuma mandarin) by nutrient competition^16,17^. A complex and large mutant library with a high insertion frequency aids in precisely identifying regions of interest^18^. Hence, we analyzed 2,804 Tn mutants of JBC17WT and identified genes, including exopolyphosphatase (*ppx*) and Xaa-Pro aminopeptidase (*pepP*), which are involved in nutrient competition^17^. Our previous results strongly suggested the role of nutrient competition in the biocontrol ability of JBC17WT. However, because of the genome sequence of 6.85 Mbp with 6,049 coding sequences of JBC17WT (NCBI Acc no. CP029693.1), likely, other genes that are important for nutrient competition in the biocontrol activity of JBC17WT could not be determined because of the low coverage of the previous Tn mutant library.

In this study, using the strain JBC17WT, which is whole genome sequenced and well-defined for nutrient competition, a new mutant library was generated using Tn systems to have a near-saturation coverage of whole genome of the bacteria. We screened the mutants for changes in conidial germination inhibition ability and biocontrol efficacy and identified genes contributing to nutrient competition. The results of this study indicated that depletion of overall nutrients by JBC17WT was crucial for the inhibition of conidial germination on nutrient-limited fruit surfaces, which consequently decreased the chances of fungal spores infecting fruits. The functions of each gene identified for conidial germination inhibition and biocontrol performance of JBC17WT require further study, which will consequently contribute to the development of biocontrol applications.

## Results

### Selection of Tn mutants exhibiting variation in inhibition activity against conidial germination

**Figure 1:**
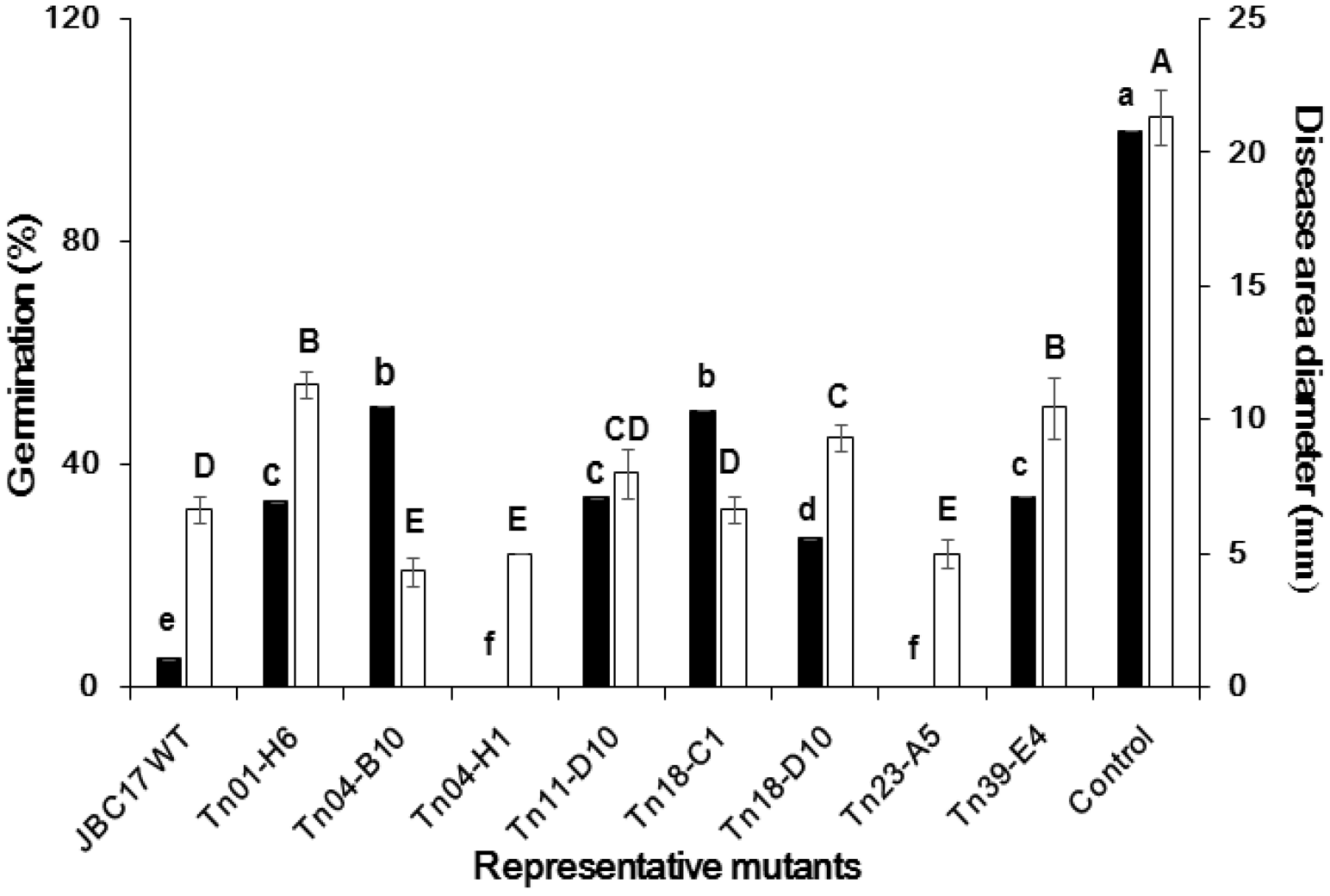
Effect of Tn mutation on conidial germination and biocontrol activity of *Pseudomonas putida* JBC17 and its Tn mutants. For the conidial germination inhibition assay, the conidia of *Penicillium digitatum* were incubated with *P. putida* JBC17 (JBC17WT) or each mutant strain, and germination was observed with a light microscope 24 h after incubation at 25 °C. For the biocontrol assay, mandarin fruits were wound-inoculated with a conidial suspension of *P. digitatum* followed by treatment with bacterial suspensions of JBC17WT or its mutants. The disease area (mm) was assessed 7 d after incubation at 20 °C and 90% relative humidity. The data represent the mean ± standard deviation, and bars with the same letter(s) do not differ significantly at P < 0.05.

In order to identify and characterize the genes involved in the inhibitory activity of JBC17WT against conidial germination of *P. digitatum*, we generated 7,002 mutants through random Tn insertion mutagenesis and the mutants in the library were individually assayed for changes in conidial germination inhibition ability. The conidial germination rate increased more than twofold in 44 mutant strains compared to the wild type, indicating a severe decrease in the ability for condial germination inhibition of the strain by the Tn insertion. The mutants were grouped into three classes according to the degree of reduction in germination inhibition activity: severe decrease (a decrease > 6.0-fold in germination rate, —), moderate decrease (a decrease < 6.0 but ≥ 4.0-fold, –), and mild decrease (a decrease < 4.0 but ≥ 2.0-fold, -) (See Supplementary Table S1 and S2 online). For instance, conidial germination by Tn01-F4 (33.3% germination rate), Tn01-H6 (50.3%), Tn11-D10 (34.0%), and Tn18-C1 (49.7%) were decreased by more than sixfold compared to that of the wild type (5.0%) (Fig. 1). Tn19-E7 (27.3%), Tn27-F8 (25.3%), and Tn54-F11 (30.0%) exhibited moderate decreases, whereas Tn05-B7 (18.3%) and Tn09-D3 (11.7%) showed a mild decrease. Surprisingly, no conidia were germinated by the nine mutants, which resulted in 100% inhibition of conidial germination (See Supplementary Table S1 online). For example, conidial germination by mutant strains, such as Tn04-H1 and Tn23-A5, was completely suppressed (Fig. 1). Our mutant library analysis was useful for identifying individual gene mutants that influenced the germination inhibition of JBC17WT.

### Functional category of genes inducing changes in the ability to inhibit conidial germination

To determine the Tn insertion site in the mutants showing variation in germination ability, we performed semi-degenerate PCR and sequencing. Among 53 mutants that showed alterations in the ability of conidial germination inhibition, insertion sites in 48 mutants were successfully identified (See Supplementary Table S1 and S2 online). The identified genes were classified based on orthologous groups in the cell (See Supplementary Table S3 online), which belong to several categories: 13 genes (*aroA*, *dgdA-*3, *pepP*, *IlvC*, and others) were involved in amino acid transport and metabolism, four genes (*fliH*, *fliR*, and *fliG*) in cell motility, two genes (*gltA* and *ctaD*) in energy production and conversion, and one gene (*FAA1*) in secondary metabolite biosynthesis, transport, and catabolism. Among the gene-identified mutants, three mutant strains (*fliR*, *cirA*, and *IlvC*) had insertion in the same open reading frame (ORF), and the mutant strains in the same gene exhibited very similar phenotypes, such as colony morphology and growth rate, irrespective of the insertion site. Among the altered mutants, three strains had Tn insertions in the intergenic region. Southern blot analysis indicated that none of the 20 randomly selected mutants had double inserts. The analyzed mutant strains and their identified genes could be a valuable resource for elucidation of the mechanisms of nutrient competition.

### Biocontrol activity of the mutants showing a decreased ability to inhibit conidial germination

**Figure 2:**
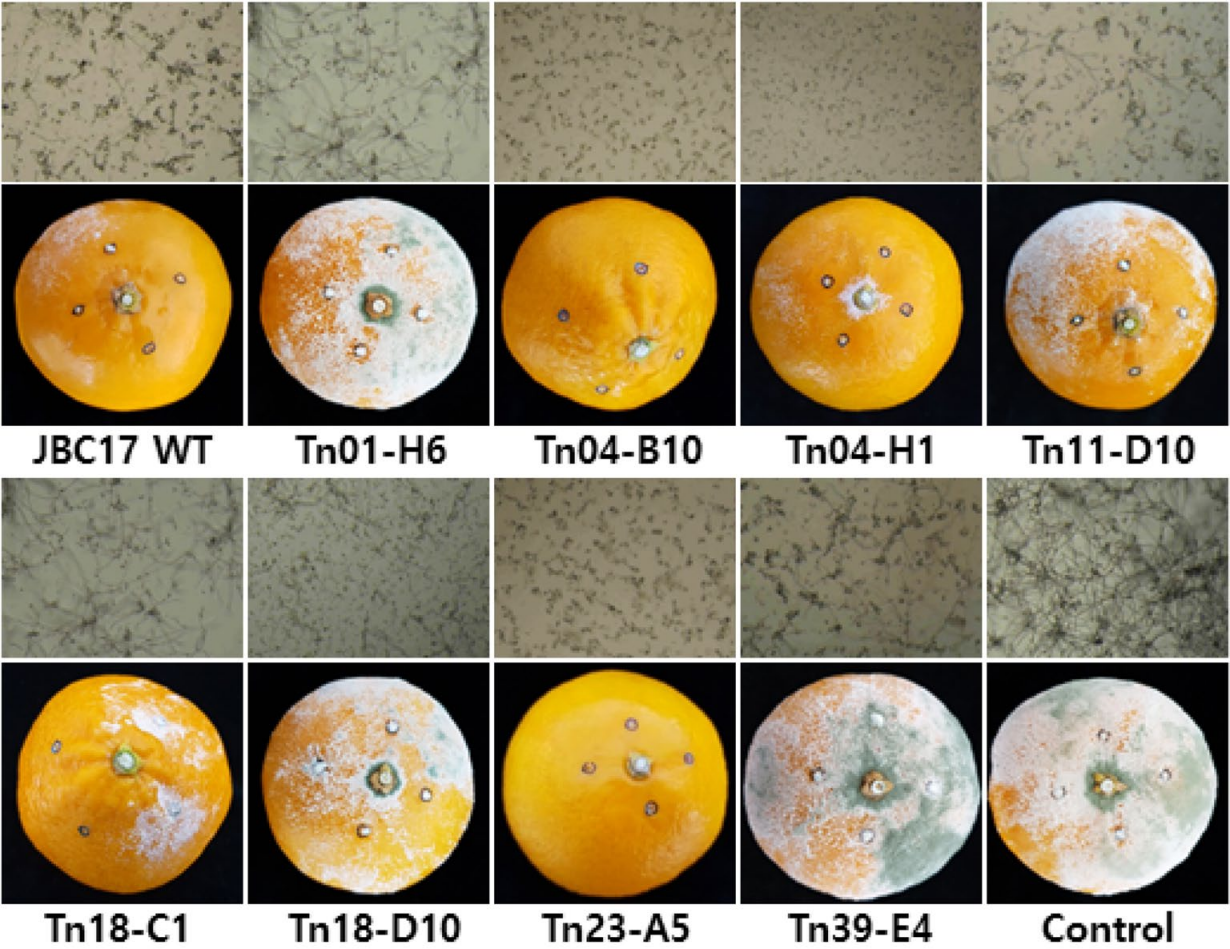
Inhibition of *Penicillium digitatum* conidial germination and suppression of green mold of mandarins by treatment with *Pseudomonas putida* JBC17. For the conidial germination assay, conidia of *P. digitatum* were incubated in 10% PDB with cells of *P. putida* JBC17WT or each mutant and were separated by a PTFE membrane. The conidial germination was photographed with a microscope after 24 h incubation at 25 °C (top panel). For the biocontrol assay, mandarin fruits inoculated with *P. digitatum* (10^6^ conidia/ml) were treated with bacterial suspensions (1 × 10^8^ cfu/ml) and then incubated at 20 °C and 90% relative humidity for 7 d (bottom panel). The figure shows representative mutants with altered activity in the inhibition of conidial germination and disease development compared to the wild type.

Conidial germination of fungal pathogens is crucial to the infection of postharvest fruits and vegetables. The JBC17WT strain and its Tn mutants suppressed the incidence of green mold by inhibiting conidial germination of the causal pathogen *P. digitatum*. To assess the contribution of each mutated gene to the biocontrol capacity of JBC17WT, each mutant strain was evaluated for its biocontrol efficacy against green mold in mandarins. In the initial stage of infection, disease development was suppressed by treatment with mutants, such as Tn11-D10 (Δ*cirA*), Tn19-E7 (Δ*serA*), and Tn01-H6 (Δ*ppx*), which showed reduced conidial germination inhibition activity. However, the disease symptoms developed rapidly from 7 d after inoculation compared to the wild type (Figs. 1 and 2). The results indicated that the suppression of conidial germination inhibition ability by mutations reduced the biocontrol capability of individual mutants in the mandarin fruits, suggesting the importance of conidial germination inhibition for the suppression of green mold disease in postharvest fruits.

### Biocontrol activity of the mutants showing an increased ability to inhibit conidial germination

In our conidial germination inhibition assay, nine mutants completely suppressed conidial germination, which indicated an increase in conidial germination inhibition by the mutation of individual genes compared to the wild type. Among them, many genes were involved in flagellar biogenesis (See Supplementary Table S1 and S2 online). Genes *fliH* (Tn04-B10) and *flgG* (Tn30-A7) are reported to be involved in flagellar assembly and biosynthesis^19,20^, *fliR* (Tn04-H1 and Tn43-A12) is part of flagellar export apparatus^21^, and RNA polymerase sigma factor *fliA* (Tn23-A5) regulates late flagellar and chemotaxis genes^22^. In addition to flagella-related genes, other genes were identified by the increase in germination inhibition. The function of *ytcJ* (Tn09-B3) and *yaiI* (Tn44-B4) remain to be uncovered, and the genes of two mutants (Tn44-G12 and Tn50-G7) were not identified (See Supplementary Table S1 online).

The effects of individual genes that increased the ability for conidial germination inhibition for biocontrol efficacy were analyzed using the mutant strains on mandarin fruits. The biocontrol activity of the mutants, such as Tn04-B10 (Δ*fliH*), Tn04-H1 (Δ*fliR*), and Tn09-B3 (Δ*ytcJ*), significantly increased compared to that of the wild type (> 90%) (Figs. 1 and 2, See Supplementary Table S1 online). Fruits treated with Tn04-H1 (Δ*fliR*) and Tn09-B3 (Δ*ytcJ*) created a water-soaked appearance without typical symptoms of green mold, and Tn04-B10 (Δ*fliH*) showed no sign of infection. The results of the biocontrol assay indicated that the increase in the ability for conidial germination inhibition increased the biocontrol activity of the mutant strains. A previous study^23^ reported that removal of flagellar machinery improved growth and biomass of *P. putida* KT2440 and tolerance against endogenous oxidative stress and survival in the stationary phase compared to the wild type. Collectively, these results indicate that flagella deficiency increased biocontrol activity and nutrient absorption ability compared to that of the wild type.

### Nutrient competition ability of the mutants

**Figure 3:**
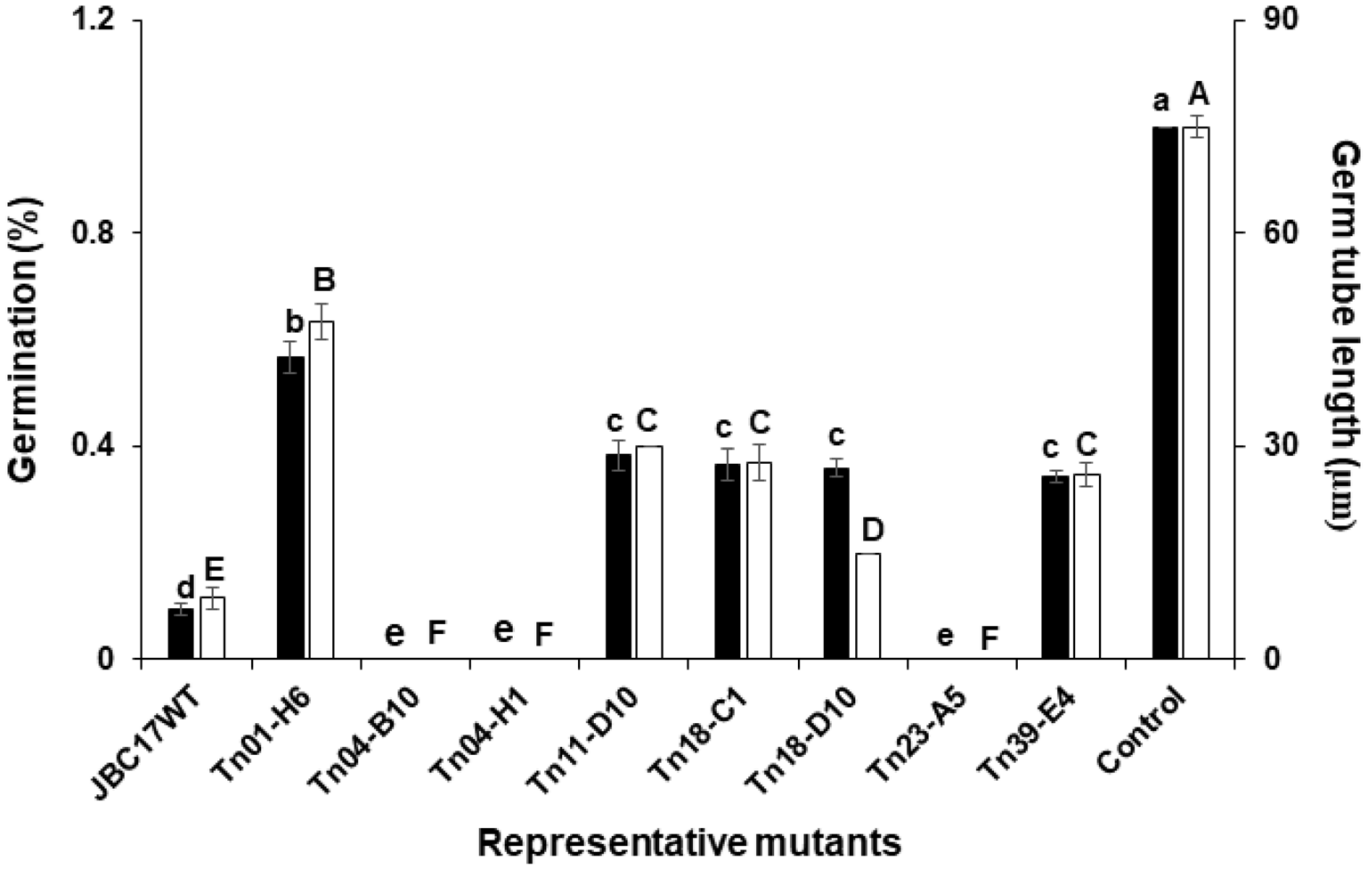
Germination of *Penicillium digitatum* conidia on PTFE membranes in cylinders. Conidia were incubated with *Pseudomonas putida* JBC17 (JBC17WT) or its Tn mutants in 10% potato dextrose broth, and germination percentage (filled square) and germ tube length (open square) were recorded 24 h after incubation at 25 °C. The number of germinated conidia from 100 observations in a microscopic view was counted, and percentage germination was compared with the wild type. The data represent the mean ± standard deviation of the replications. The bars with the same letter do not differ significantly at P < 0.05 according to Tukey’s test.

To understand the mechanisms that inhibited spore germination, we analyzed the nutrient competitive ability of the strains using a polytetrafluoroethylene (PTFE) membrane. The conidial germination rate and germ tube length were significantly reduced to 9.3% and 8.67%, respectively, by co-incubation with JBC17WT (Fig. 3). The mutants Tn04-B10 (Δ*fliH*), Tn04-H1 (Δ*fliR*), Tn09-B3 (Δ*ytcJ*), and Tn23-A5 (Δ*fliA*) completely inhibited germination of *P. digitatum* conidia. The germination percentages of Tn01-H6 (Δ*ppx*) and Tn11-D10 (Δ*cirA*) were 56.7% and 38.3%, respectively, followed by Tn18-C1 (Δ*pepP*, 36.7%), Tn18-D10 (Δ*argG*, 36.0%), Tn19-E7 (Δ*serA*, 29.0%), and Tn39-E4 (Δ*dnaJ*, 34.3%). Many mutated genes that induced decrease in activity for conidial germination inhibition were involved in the transport and metabolism of organic and inorganic compounds (See Supplementary Table S1 online). An additional 24 h incubation of the PTFE chamber in fresh 10% PDB without bacteria induced more than 90% conidial germination from all tested mutants, indicating no fungicidal antagonism of the bacterial strain against conidial viability during contact. The results indicate that the wild type and mutants deprive nutrients of the conidia at nutrient-limited fruit surfaces, thereby reducing germination and consequently disease incidence.

### Growth rate of the mutants

**Figure 4:**
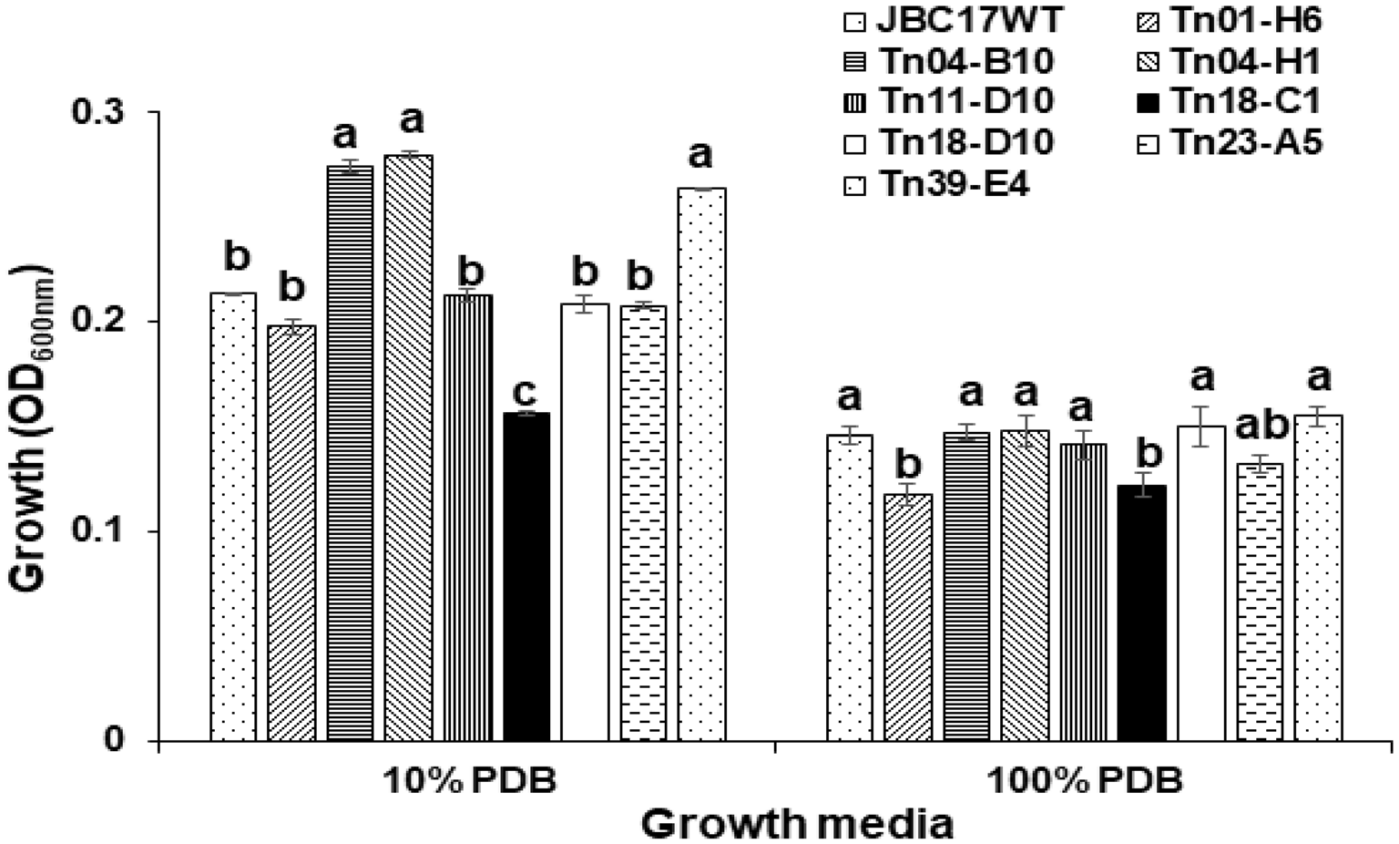
Comparison of growth rate of *Pseudomonas putida* JBC17 and its mutants in nutrient-rich and -poor media. The bacterial cells (OD_600_ = 0.05) were inoculated into the fresh nutrient-limited (10% PDB) and -rich (100% PDB) media and incubated at 28 °C with shaking at 180 rpm. Growth was compared to that of the wild type 12 h and 24 h after incubation for 100% PDB and 10% PDB, respectively. The data represent the mean ± standard deviation of the replications, and the same letters indicate a non-significant difference at P < 0.05 according to Tukey’s test.

The results of this study indicated that gene mutations could influence the nutrient absorption ability of JBC17WT from the surrounding environment. To assess whether nutrient absorption ability was related to differences in growth of the bacteria, the growth rate of mutants in nutrient-poor (10% PDB) and nutrient-rich (100% PDB) media were compared to that of the wild type. In nutrient-rich media, the growth rate of the flagella-related mutants, Tn04-B10 (Δ*fliH*), Tn04-H1 (Δ*fliR*), and Tn23-A5 (Δ*fliA*), was similar to that of the wild type. However, under nutrient-poor conditions, the growth rates of the mutants were more than 20-fold higher than those of JBC17WT (Fig. 4). Martínez-García et al.^23,24^ reported that the growth of a flagella-deficient mutant of *P. putida* KT2440 was not significantly different in the LB medium, but the growth was higher in M9 minimal medium with fructose as the carbon source. Collectively, these results indicate that flagella depletion increases the growth rate of bacteria under nutrient-limited conditions. The growth rates of other mutant strains were similar to those of JBC17WT under nutrient-limited conditions (10% PDB), except for the mutant Tn18-C1 (Δ*pepP*), which exhibited the lowest growth rate (Fig. 4). In nutrient-rich media (100% PDB), the growth of most mutants was similar to that of the wild type, but the growth of Tn01-H6 (Δ*ppx*) and Tn18-C1 (Δ*pepP*) was reduced compared to the wild type, which requires further investigation to determine the underlying reasons. Overall, the results indicated that deficiency of flagella formation by the mutations in flagella-related genes might reduce the metabolic energy of the mutant strains, which instead induces an increase in growth rate. The rapid proliferation of bacterial cells at the fruit surface might increase the competitiveness of the bacteria for nutrients and niches.

### Carbon source utilization pattern of the mutants

**Figure 5:**
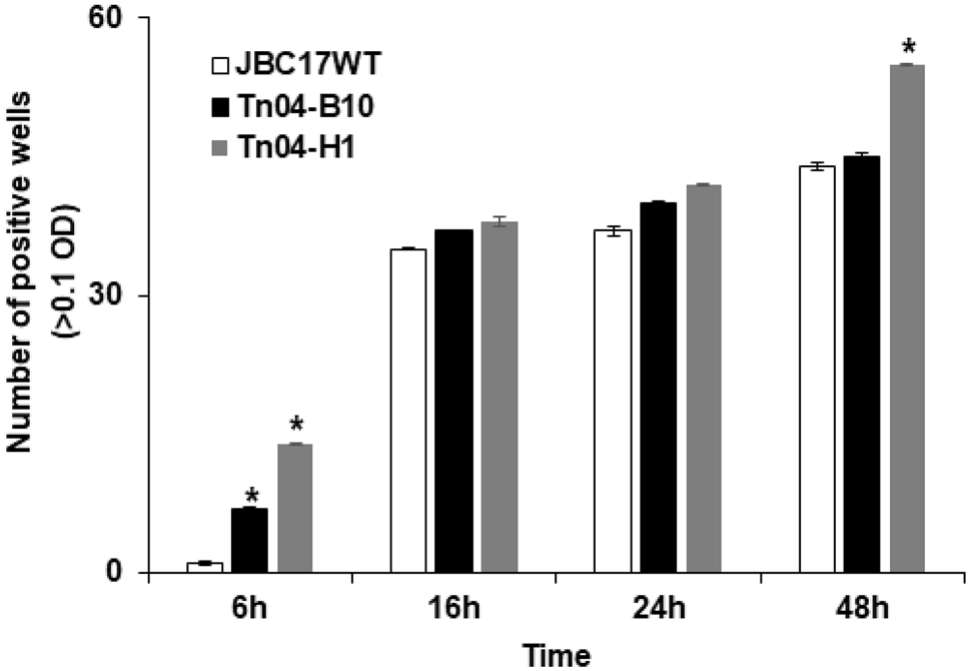
Number of carbon substrates utilized by *Pseudomonas putida* JBC17 and its flagella-related mutants. The number of wells that show color development by flagella-deficient mutant, Tn04-B10 (*fliH*) and Tn04-H1 (*fliR*), were assayed using Biolog GN2 microplate at 6, 16, 24, and 48 h after incubation and compared to the wild type. Data are presented as the mean ± standard deviation of three replicates, and values marked by an asterisk (*) are significantly different at each time point at P < 0.05 according to Student’s t-test.

The Tn mutation altered the growth rate or metabolism of wild type, which could induce changes in the utilization of nutrient sources by the bacteria. The average color development (AWCD) of 95 carbon sources was assessed from 6 to 48 h after incubation to compare the carbon utilization patterns of the flagella-related mutants and wild-type strains. In the plates with mutants Tn04-B10 (Δ*fliH*) and Tn04-H1 (Δ*fliR*), color developed in 7 and 14 substrates, respectively, at 6 h after incubation, whereas only one substrate was utilized by JBC17WT (Fig. 5). At 16 h and 24 h, there was no significant difference in the number of substrates utilized between the mutants and the wild type. After 48 h of incubation, the AWCD showed continuous utilization of substrates by the Tn04-H1(Δ*fliR*) strain. Overall, the nutrient utilization by flagella-related mutants, Tn04-B10 (Δ*fliH*) and Tn04-H1 (Δ*fliR*), started at an early incubation time, and they continued to utilize more nutrients than the wild type to at least 48 h after incubation.

**Figure 6:**
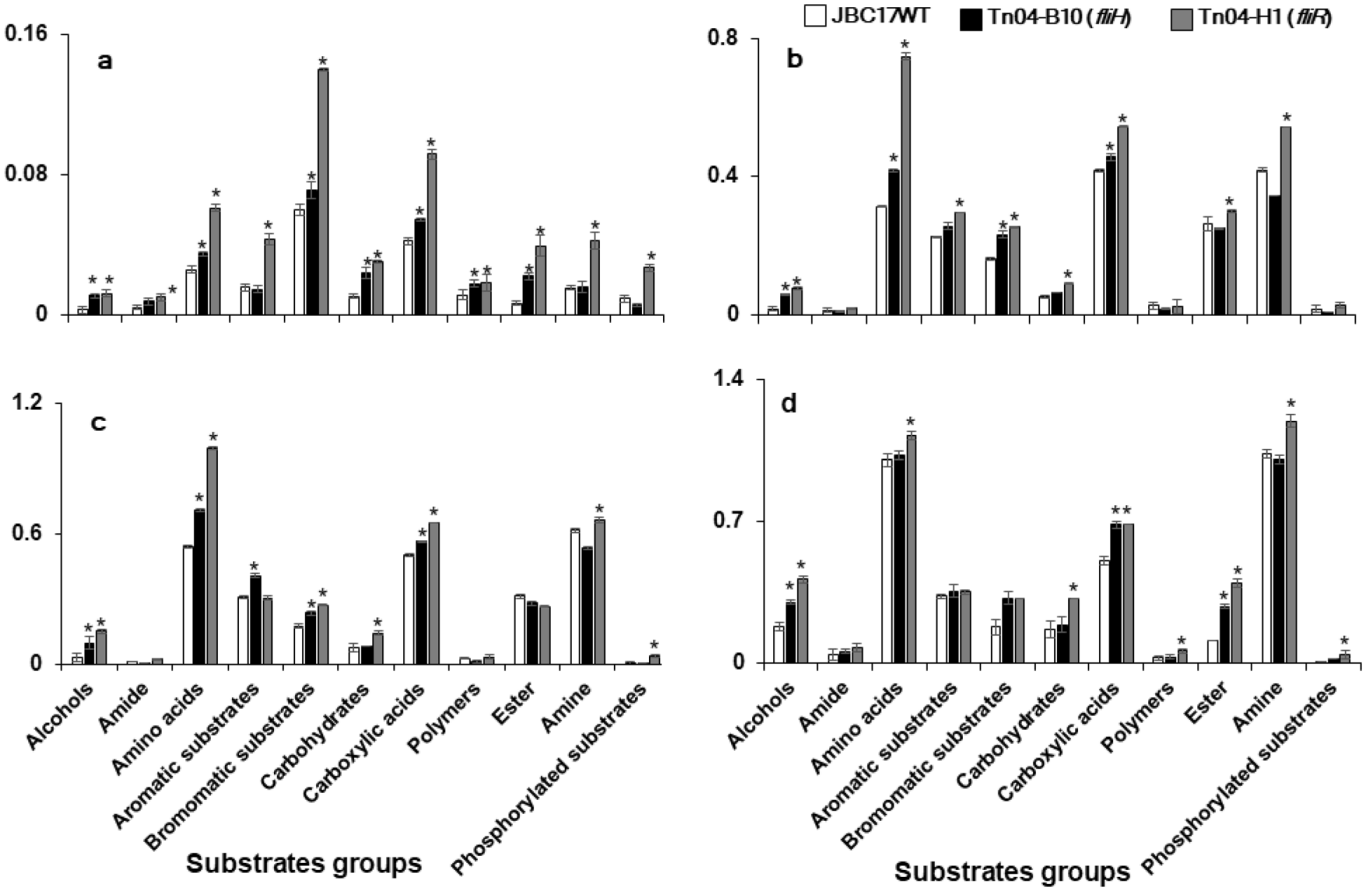
Average well color development (AWCD) and utilization of various groups of carbon substrates by *Pseudomonas putida* JBC17 and flagella-related mutants. *P. putida* JBC17 (JBC17WT) and its flagella-deficient mutants, Tn04-B10 (*fliH*) and Tn04-H1 (*fliR*), were incubated in Biolog GN2 microplate. AWCD of 95 carbons were recorded at OD_600_ at 6 h (**a**), 16 h (**b**), 24 h (**c**), and 48 h (**d**) and compared to the wild type. Data are presented as the mean ± SD of three replicates, and values marked by an asterisk (*) are significantly different from the wild type in absorbance at P < 0.05 according to Student’s t-test.

Color development in each utilized carbon substrate was different between the mutant strains and the wild type. The AWCD of Tn04-B10 (Δ*fliH*) was higher than that of the wild type, except for amides, amines, aromatic substrates, and phosphorylated substrates at 6 h after incubation. Additionally, Tn04-B10 (Δ*fliH*) continuously consumed amino acids, carbohydrates, carboxylic acids, and amines at a higher rate than the wild type up to at least 48 h after incubation. The overall AWCD in Tn04-H1 (Δ*fliR*) was significantly higher than that in JBC17WT at 6 h after incubation (Fig. 6a), and the utilization of amino acids, carbohydrates, carboxylic acids, and amines was significantly higher than that in the wild type from 16 to 48 h after incubation (Fig. 6b-d). Overall, our results indicated that flagella-related mutants absorb nutrients, such as amino acids and amines, quickly and continuously for rapid growth, which could consequently deplete nutrients required for spore germination on nutrient-limited fruit surfaces.

### Swarming and swimming motility of the mutants

**Figure 7:**
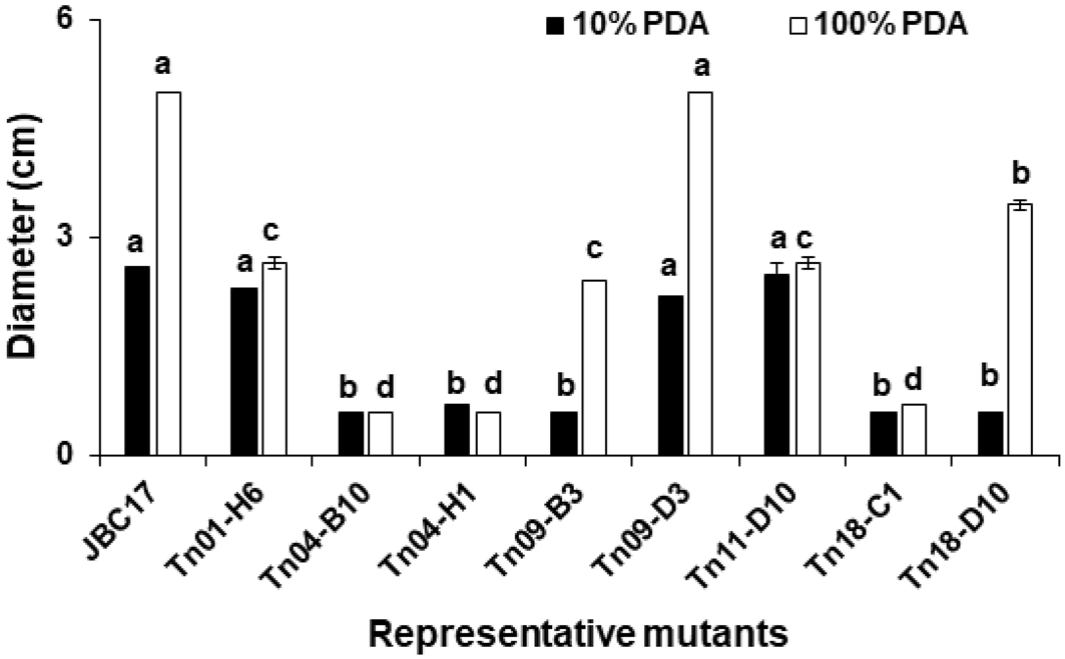
Effect of mutations on swimming motility of *Pseudomonas putida* JBC17 and its mutants. Each strain was grown overnight in LB broth and inoculated in the center of nutrient-poor (10% PDA) and -rich (100% PDA) soft agar (0.3% agar) plates. The colony diameter was recorded 48 h and 24 h after incubation for 10% and 100% PDA, respectively.

**Figure 8:**
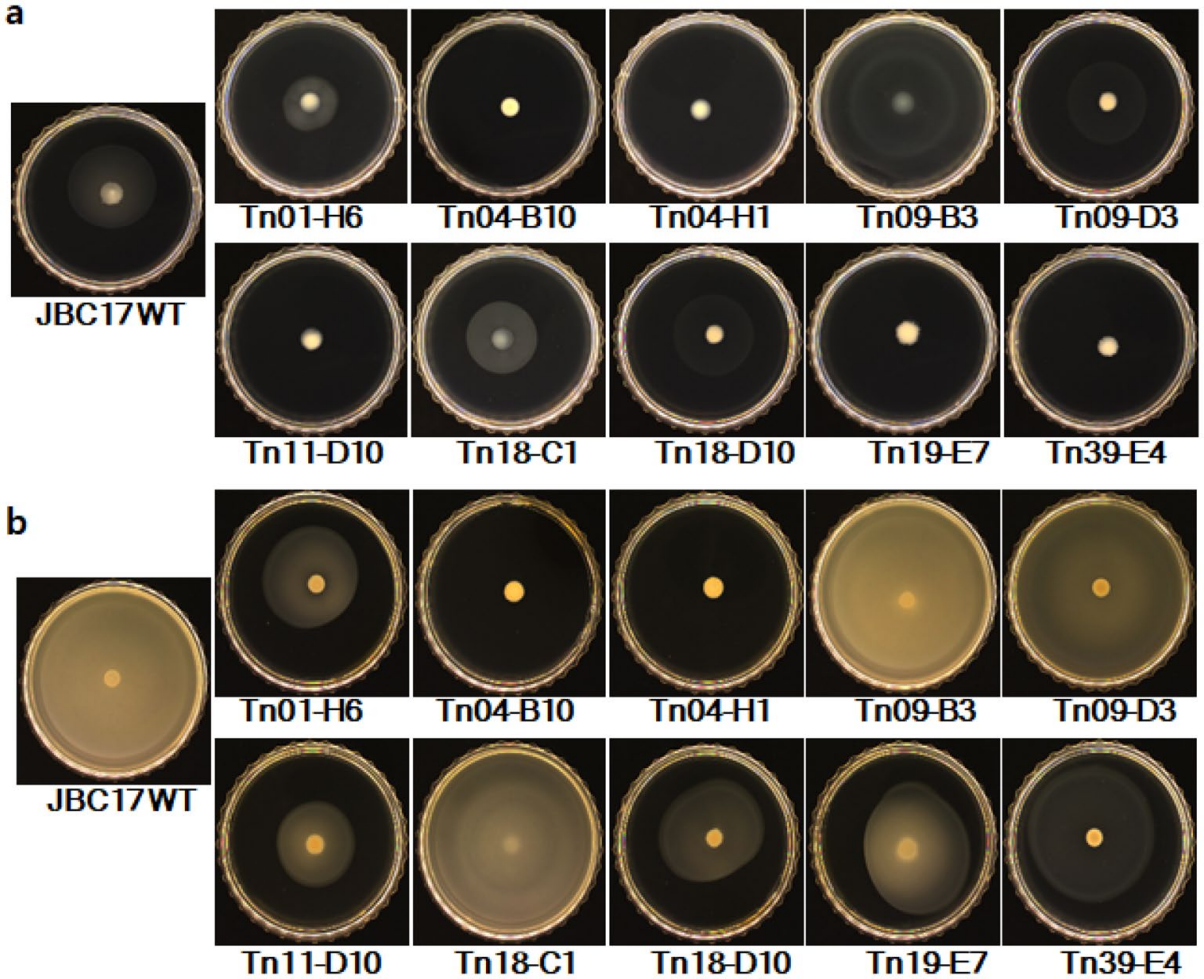
Effect of mutations on swimming motility of *Pseudomonas putida* JBC17 and its mutants. Each strain was grown overnight in LB broth and inoculated in the center of nutrient-poor 10% PDA (**a**) and -rich 100% PDA (**b**) containing 0.3% agar and incubated for 48 h and 24 h, respectively.

Motility is important for the survival of bacteria and influences the biocontrol performance of BCAs^25^. To investigate the influence of mutation on motility and biocontrol efficacy, swarming (0.5% agar) and swimming (0.3% agar) motility was assayed. Strain JBC17WT and all of the Tn mutants failed to move in soft PDA plates (0.5% agar), indicating no swarming motility irrespective of nutrient-poor and nutrient-rich conditions (See Supplementary Fig. S1 online). When we assessed swimming motility by reducing the agar percentage (0.3%), JBC17WT moved quickly, whereas the flagella-related mutants, Tn04-B10 (Δ*fliH*) and Tn04-H1 (Δ*fliR*), significantly decreased motility irrespective of the nutrient concentration of the medium (Figs. 7, 8). The mutant strain Tn01-H6 (Δ*ppx*) showed a mild reduction in swimming motility, whereas Tn18-C1 (Δ*pepP*) motility was similar to that of the wild type, irrespective of nutrient conditions. The swimming motility of Tn09-B3 (Δ*ytcJ*) increased under nutrient-rich and nutrient-poor conditions (Fig. 8a, b), the mechanism of which requires further study. The motility of mutant Tn39-E4 (Δ*dnaJ*) and Tn11-D10 (Δ*cirA*) was significantly suppressed, and the swimming motility of Tn18-D10 (Δ*argG*) and Tn19-E7 (Δ*serA*) was slightly reduced compared to that of the wild type. In *P. aeruginosa*, disruption of *serA* affects swimming, swarming motility, and adherence^26^. Overall, the results indicated that the rapid growth of BCAs at nutrient-limited infection sites increases the biocontrol activity of JBC17WT, whereas swarming motility was not significant for the strain to compete at the small infection sites of fruits.

### Recovery of inhibition ability and phenotypes by *fliR* gene complementation

**Figure 9:**
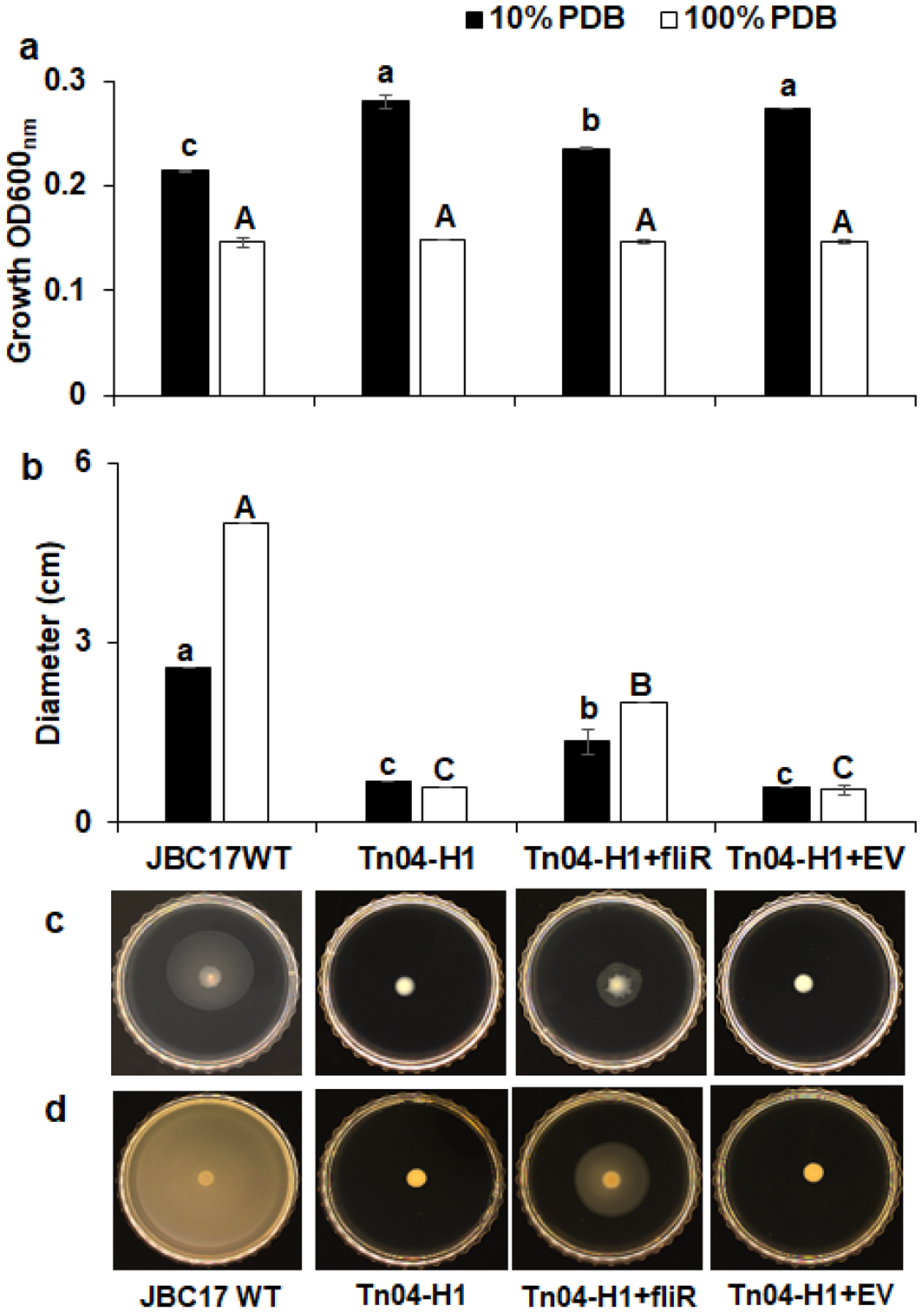
Complementation of *fliR* gene into Tn mutant strain Tn04-H1 of *Pseudomonas putida* JBC17. The gene was cloned into pUCP18*Cat* and transformed into the *fliR* mutant strain (Tn04-H1) to produce the complemented strain (Tn04-H1 + *fliR*). An empty vector (EV) was also transformed into the mutant to produce a control strain (Tn04-H1 + EV). (**a**) In vitro growth in 10% and 100% PDB; (**b**) Swimming motility in (**c**) nutrient-poor 10% PDA and (**d**) nutrient-rich 100% PDA containing 0.3% agar. Vertical bars indicate the mean ± SD of the replications. Bars with the same letters are not significantly different at P < 0.05 according to Tukey’s test.

To assess the influence of flagella production on the phenotypes and the ability to inhibit spore germination of Tn04-H1 (Δ*fliR*), we complemented the mutant strain with plasmids containing the *fliR* gene from the wild type. We compared the growth rate, swimming motility, and ability to inhibit germination with those of the wild type. There was no significant difference in growth between the strains under nutrient-rich conditions (100% PDB). However, the growth of the complemented strain (Tn04-H1 + *fliR*) was significantly lower than that of the mutant Tn04-H1 (Δ*fliR*) but higher than that of JBC17WT in 10% PDB (Fig. 9a). The complementation of the *fliR* gene into the mutant strain marginally recovered the swimming motility of the strain (Fig. 9b-d) in nutrient-poor and nutrient-rich media. Transformation with an empty vector in the mutant Tn04-H1(Δ*fliR*) did not affect the growth and swimming motility of Tn04-H1 (Δ*fliR*) compared to JBC17WT. The complemented strain completely inhibited conidial germination under nutrient-poor (10% PDB) conditions, similar to the mutant strain Tn04-H1 (Δ*fliR*). The production and function of flagella are complex processes that require coordination in space and time^27^. The complementation of the mutated gene with the plasmid may not restore all the alterations induced by the mutation. Overall, complementation of the *fliR* gene marginally recovered swimming motility and growth rate, although it was still faster than the wild type. The results of the complementation assay indicated that rapid growth because of the flagella deficiency contributed to the ability to inhibit conidial germination of JBC17WT.

## Discussion

Antagonism, secretion of hydrolytic enzymes, induced systemic resistance, and competition for nutrients and niches are well-known mechanisms for the biocontrol of plant diseases^3,28,29^. Competition for nutrients and space plays a crucial role in postharvest disease control of vegetables and fruits^9,30^. To be an effective BCA, a strain must be able to colonize plant surfaces in nutrient-deficient conditions at population densities sufficient to produce a beneficial effect. Some beneficial microorganisms colonize the damaged fruit by assimilating the carbon sources for survival and proliferation, thereby depleting carbohydrates and starving the competing pathogens^30^. Additionally, many BCAs compete for limited nitrogen sources, such as amino acids, or iron by siderophore production, which contributes to the antagonistic potential of the bacteria^7,14^. However, the intrinsic mechanism and contribution to the biocontrol activity by nutrient competition and the key nutrient compounds for competition between bacterial BCAs and fungal pathogens remain to be elucidated. In our previous studies, JBC17WT suppressed the blue mold and green mold of mandarins by nutrient competition^17^. In that study, we analyzed 2,804 Tn mutants of JBC17WT and identified several genes involved in inhibiting conidial germination. However, the number of analyzed mutants has to be increased to cover most of the coding genes of JBC17WT, and the contribution of each gene to biocontrol activity and inhibition of conidial germination required further analysis. In this study, genes essential to inhibiting conidial germination were identified by screening newly constructed Tn mutants, and their contributions to overall biocontrol efficacy were evaluated. The present study revealed many new genes that affecting conidial germination and biocontrol activity of JBC17WT by nutrient competition.

In our analysis, many mutants that were impaired regarding genes related to flagella formation showed increased efficacy in biocontrol ability and inhibition of conidial germination compared to JBC17WT. The mutants Tn04-B10 (Δ*fliH*) and Tn30-A7 (Δ*flgG*) were impaired in genes involved in flagellar assembly and biosynthesis, respectively^19,20^. Both Tn04-H1 (Δ*fliR*) and Tn43-A12 (Δ*fliR*) had mutations in the *fliR* gene, which is a part of the flagellar export apparatus^21^. Tn23-A5 (Δ*fliA*) had a Tn insert in the RNA polymerase sigma factor regulating late flagellar and chemotaxis genes^22^. The mutant strains that were impaired in flagella-related genes were significantly compromised in swimming motility in nutrient-rich and nutrient-poor conditions. Biofilm formation is a complex phenomenon involving adhesion proteins, flagella, and exopolysaccharides^22^. Gjermansen et al.^31^ reported that *P. putida* cells underwent rapid dispersal under low nutrient conditions, whereas flagella-lacking mutants persisted as biofilms. Flagella of *Bacillus subtilis*^32^ and *Vibrio cholerae*^33^ were important for biofilm formation, allowing the bacteria to swim and to detect a surface. In our analysis, although the lack of motility and biofilm formation of the flagella-deficient mutants were not strictly correlated, biofilm formations of Tn04-B10 (Δ*fliH*) and Tn04-H1 (Δ*fliR*) were decreased compared to wild type. However, we observed that cells of mutants impaired regarding flagella-related genes tended to be densely flocked together in liquid culture, whereas they were evenly suspended in the case of JBC17WT cells. During the motility assay, the colonies of the mutants under nutrient-limited conditions showed a dense accumulation of cells at the center of the colony where bacterial cells were inoculated, and colonies spread slightly outward after 48 h of growth. The attachment of the mutant cells might be due to exopolysaccharide production. Recently, flagellar mutants of *Pseudomonas aeruginosa* overproduced exopolysaccharide and showed increase of biofilm formation, which providing a fitness benefit to the bacteria^34^. We need further exploration to understand influence of flagella on exopolysaccharide production and adhesion of JBC17WT.

Bren et al.^35^ reported that bacterial cells apply a strategy to prolong the exponential stage to maintain their growth rate under nutrient-limiting conditions during batch culture. The flagella-lacking *P. putida* mutant grew well in fructose-amended M9 medium compared to the wild type^24^. In our study, most of the Tn mutants, irrespective of mutated genes, showed similar growth rates in nutrient-rich media. However, mutants of flagella-related genes grew faster than JBC17WT in nutrient-limited media, indicating that disfunction of flagellar apparatus led to faster growth. The faster and higher quantity of sugar utilization by yeast led to the inhibition of *Botrytis cinerea* conidial germination^36^. A non-flagellated strain of *P. putida* KT2440 showed advantages in growth rate, adaptation, and resistance to stresses compared to the motile wild type^23^, which is consistent with our results. Mutants of *Pseudomonas putida* KT2442 deleting the genes involved in the biosynthesis and assembly of flagella and pili grew faster and showed sharply decreased biofilm, but accumulated more polyhydroxyalkanoate^37^. Taken together, the increase in growth rate might be advantageous for the mutants in establishment and colonization on the fruit surfaces, thereby out-competing the conidial germination of the fungal pathogen.

Regulation of motility-related genes is dependent on nutrient status^38^. Carbon source utilization influences the activity of phytopathogens in the presence of different root exudates^39^. Changes in growth rate can induce changes in nutrient utilization rates and patterns, which alter the nutrient absorption sources of the mutants. In this study, the carbon utilization patterns of Tn04-B10 (Δ*fliH*) and Tn04-H1 (Δ*fliR*) were compared to those of JBC17WT using Biolog plates. Tn04-H1(Δ*fliR*) utilized carbohydrates and amino acids faster and longer than did JBC17WT. The nutrient utilization patterns of the mutants in flagella-related genes were similar. In citrus fruits, nearly 70% of the total nitrogen is present in the form of amino acids. The quick and continuous absorption of nitrogen nutrients from the environment might be advantageous for the mutants in outcompeting the pathogen at the infection site. Therefore, in this study, the increase in amino acid and carbohydrate utilization by the mutations affected biocontrol performance via changes in the ability to inhibit conidial germination. *P. putida* KT2440 senses competition and indirectly starves the competing species by increasing metabolic function to rapidly utilize available carbon sources and multiply quickly, which starves the competing species by nutrient absorption^40^. In summary, rapid growth without movement along with rapid utilization of nutrients, such as amino acids, by flagella-related mutants might lead to nutrient depletion at the site of infection, which eliminates the pathogen. The key compounds for nutrient competition in the wounds of mandarin fruit surfaces remain to be revealed.

A mutation in *cirA* (Tn11-D10 and Tn25-H10) reduced conidial inhibition ability, and consequently, suppressed biocontrol activity. Gene *cirA*, a TonB-dependent transporter in the bacterial outer membrane, transported siderophores^41^. TonB-dependent receptors help Gram-negative bacteria take up scarce nutrients during competitive interactions, and they influence the uptake of sugars, vitamins, and other non-ferrous cations^42^. Thus, the mutation in *cirA* reduces nutrient utilization of BCAs at the infection sites, which consequently reduces biocontrol activity. The *serA* (Tn19-E7) gene catalyzes the reversible oxidation of 3-phospho-D-glycerate to 3-phosphonooxypyruvate, the first step in the phosphorylated L-serine biosynthesis pathway in *E. coli* and *Corynebacterium glutamicum*^43,44^. L-serine and L-aspartic acid have been reported to enhance the biocontrol activity of *Candida sake* CPA-1 against *Penicillium expansum* in apples^45^. The mutation in *serA* might reduce the biocontrol ability of JBC17WT because of the low availability of L-serine. The *argG* (Tn18-D10) gene encodes argininosuccinate synthase, an arginine biosynthetic enzyme in *Pseudomonas*^46^. Mutation in the *argG* gene led to reduced fitness of the *P. aeruginosa* strain PGPR2 during corn root colonization^47^, whereas a mutant of the *P. fluorescens* strain WCS365 auxotrophic for arginine had reduced root-colonizing ability in tomatoes^48^. These previous reports indicated the importance of arginine in surface attachment, which is likely crucial for biocontrol. Thus, the mutation in *argG* might indirectly influence the biocontrol ability of JBC17WT by reducing its ability to utilize nutrients and establish at the site of application. However, further studies are needed to establish the role of these genes in the biocontrol ability of BCAs.

The genes *ppx* (Tn01-H6) and *pepP* (Tn18-C1) were reported to play important roles in the biocontrol ability of JBC17WT in our previous study^17^. In this study, the growth of Tn01-H6 (Δ*ppx*) and Tn18-C1 (Δ*pepP*) mutants decreased irrespective of nutrient content, which indicated that the defect in nutrient usability by the mutation retarded the growth rate. The ability to inhibit conidial germination of Tn18-C1 (Δ*pepP*) was decreased, similar to our previous reports, but the biocontrol activity of the mutant was slightly decreased compared to that of the wild type. The mutant Tn39-E4 (Δ*dnaJ*) showed reduced biocontrol ability and motility, whereas growth was similar to that of the wild type, irrespective of nutrient status. The chaperone gene *dnaJ* (also known as Heat Shock Protein 40) and *dnaK* and *grpE* were involved in the regulation of putisolvin biosynthesis, a cyclic lipopeptide in *P. putida*, and also affected motility^49^. In *E. coli*, DnaKJE assists in protein folding, disaggregation, and translocation through biological membranes^50^. DnaK affects cellular susceptibility to nutrient starvation^51^ and proper functioning of the central metabolism and cellular response to alterations in carbon nutrition in *E. coli*^52^. Therefore, the mutation in *dnaJ* affects DnaK function, which leads to suppression of biocontrol activity by compromising nutrient absorption.

In this study, high-throughput Tn mutant analysis identified genes that influenced the ability to inhibit conidial germination of JBC17WT. Mutation of flagella-related genes enhanced the growth rate of JBC17WT under nutrient-limited conditions and induced rapid and continuous absorbance of carbohydrates, amino acids, and amines, which might deprive the nutrients needed for conidial germination of *P. digitatum*. Additionally, amidohydrolase (*ytcJ*), tonB-dependent receptor (*cirA*), argininosuccinate synthase (*argG*), D-3-phosphoglycerate dehydrogenase (*serA*), and chaperone protein (*dnaJ*) influenced biocontrol performance, as well as the ability to inhibit conidial germination. The identified genes will be valuable for developing antagonistic strains with higher nutrient absorption rates and increased survival and proliferation on the plant surface. The results of this study indicate that rapid and continuous absorption of nutrients by bacterial growth removes nutrient sources that are essential for conidial germination on nutrient-limited fruit surfaces, which consequently decreases the opportunity of fungal conidia to infect fruits. This study highlights the importance of nutrient competition for biocontrol activity in nutrient-limited environments. The investigation will be continued to determine the function of genes involved in the ability to inhibit conidial germination and biocontrol performance of JBC17WT.

## Materials and methods

### Culturing of JBC17WT and its mutants

Bacterial suspensions were prepared from bacteria grown in a shaking incubator at 30 °C and 250 rpm in Luria–Bertani (LB) broth. Bacterial cells were harvested at the beginning of the stationary phase by centrifugation at 4,000 × *g* for 15 min. The bacterial pellet was resuspended in 0.05 M phosphate buffer (pH 6.5) or distilled water to the desired concentration, as needed.

### Culturing of the green mold pathogen

*Penicillium digitatum* KACC42258 isolated from citrus fruits was obtained from the Korean Agricultural Culture Collection (KACC). The fungal isolate was maintained on potato dextrose agar (PDA) medium and stored at 4 °C. Mycelial plugs from the stored PDA tubes were subcultured in fresh PDA plates and incubated at 25 °C for further use. Conidial suspensions were obtained from 10 d old cultures on PDA plates by adding sterile distilled water with 0.05% tritonX-100. A sterile glass rod was used to rub the conidia off the surface of the culture, and cell suspensions were sieved through four layers of sterile cheesecloth to remove mycelial fragments. Cell numbers were counted in a hemocytometer and diluted to the optimal concentration needed for mandarin fruit inoculation or conidial inhibition assays.

### Generation of Tn insertion mutants

The random transposon mutant library of JBC17WT was constructed using Tn T8 (IS*lacZ*/hah-tc) and its conjugal delivery suicide plasmid (pIT2)^53^ using a modified biparenting mating procedure^25^ (See Supplementary Table S4 online). The plasmid pIT2, kindly donated by Dr. Gallagher’s lab^53^, was transformed by heat shock into the donor *Escherichia coli* strain MGN-617, a SM10λpir derivative forming SM10λpir/pIT2. The donor strain was grown in LB broth with ampicillin (100 µg/ml) and diaminopimelic acid (DAP 50 µg/ml) at 37 °C to an OD_600_ of ~ 1.0. The recipient JBC17WT was grown in LB broth with ampicillin (100 µg/ml) at 30 °C and 200 rpm overnight. Equal volumes (1 ml) of donor and recipient cultures were centrifuged separately, and the pellets were washed twice with LB. The pellets were resuspended in 500 µl of 10 mM MgSO_4_ and mixed. The mixture was washed twice with 10 mM MgSO_4_ and finally resuspended in 20 µl of 10 mM MgSO_4_. The suspension was then spotted onto a nitrocellulose filter (0.45 µm pore size) placed pellets side up on a pre-warmed LB agar plate containing DAP 50 µg/ml. The conjugation plate was incubated for 3 h at 30 °C. Cells were washed from the filter into 10 ml of 10 mM MgSO_4_, which was plated (200 µl aliquots) on LB agar containing tetracycline (30 µg/ml) without DAP to select against the donor strain. The plates were incubated at 30 °C until colonies formed. The colonies were picked using sterile toothpicks and individually arrayed into 96 well plates containing LB broth with tetracycline and 15% glycerol. The plates were incubated at 30 °C without shaking for 24 h and stored at − 80 °C until use in subsequent assays.

### Conidial germination inhibition assay by direct contact

The Tn insertion mutants were screened for their ability to inhibit conidial germination of *P. digitatum* following Calvo et al. (2007)^54^ with minor modifications. The mutants were revived individually from glycerol stock by growing overnight at 30 °C on LB agar plates containing tetracycline. Each strain was then incubated in LB broth overnight at 30 °C with shaking at 200 rpm and suspended in 10% potato dextrose broth (PDB). Conidia were obtained as described above. Aliquots of 100 µl of 10% PDB containing conidia (1 × 10^4^ conidia/ml) and each mutant (1 × 10^8^ cfu/ml) were placed in 96-well plates and incubated for 24 h at 25 °C. The germination of conidia was stopped with lactophenol cotton blue, and the plates were viewed under a Nikon inverted research microscope (ECLIPSE Ti2-U, Japan) to determine the germination rate. Five microscopic fields were selected, and 10 conidia were observed per field. The number of germinated conidia (the conidium was considered to have germinated when the germ tube length was equal to the length of the conidium) and the length of the germ tubes was measured with a micrometer. The percentage of inhibition was calculated by comparison with the untreated control^17^. The mutants that showed reduced or increased inhibition activity against conidial germination compared to JBC17WT were selected. Each experiment was repeated three times.

### Determination of Tn insertion site and identification of the mutated genes

Insertion sites were determined by sequencing the amplicons obtained from semi-degenerate PCR using genomic DNA of each selected mutant as a template. Each strain was incubated in LB at 30 °C, overnight cultured cells were pelleted, and genomic DNA was isolated using a commercial kit (GeneAll Exgene Cell SV mini kit, Korea) following the manufacturer’s protocol. PCR was performed as previously described^55^, with minor modifications. Transposon-specific primer LacZ-211 and an equimolar mixture of degenerate primers CEKG-2A, CEKG-2B, and CEKG-2C (See Supplementary Table S5 online) were used for the first round of PCR. The PCR conditions were as follows: 95 °C for 2 min, followed by six cycles at 98 °C for 20 s, 56 °C (-1 °C per cycle) for 20 min, 72 °C for 2 min, 25 cycles at 98 °C for 20 s, followed by 65 °C for 20 s, 72 °C for 2 min, and a final extension of 10 min at 72 °C. The first-round amplicons (1 µl) were used as templates for the second-round PCR using the transposon-specific nested primer LacZ-148 and primer CEKG-4 under the same PCR conditions, excluding the initial six cycles.

To map the transposon insertion in each mutant, the PCR product was cleaned using shrimp alkaline phosphatase and exonuclease^25,55^. The cleaned amplicons were sequenced using an Applied Biosystems BigDye Terminator v2.3 (Bionics, South Korea). The resulting sequences were analyzed to remove possible transposon sequences and aligned with the genome sequence of *P. putida* JBC17 (NCBI Acc. CP029693.1) using a BLAST analysis to identify the mutated genes. The functional category of each gene was classified based on the EggNOG database (http://eggnog.embl.de.).

### Biocontrol assay

*Penicillium digitatum* was inoculated onto mandarin fruits following Yu and Lee^17^. Briefly, the fruits, which were surface-disinfested with 70% ethanol, were wounded with a toothpick by making an injury 1 mm deep, and a 10 μl conidial suspension (10^6^ conidia/ml) of *P. digitatum* was applied to each wound, followed by inoculation with a 20 μl suspension of JBC17WT (1 × 10^8^ cfu/ml) or flagella-related mutants after 1 h. The treated fruits were placed on a water-saturated Kimwipe (Kimberly-Clark, Irving, TX, USA) in a plastic box and incubated at 20 °C and 90% relative humidity in closed plastic containers. Ten fruits with four wounds per fruit were used for each treatment, with three replicates. Data were recorded as the number of infected wounds 7 d after inoculation, and the diameter of the disease area was measured (mm).

### Nutrient competition assay using a PTFE membrane

The inhibition of conidial germination during simultaneous growth of bacterial strains and conidia of *P. digitatum* was caused by nutrient competition, as confirmed by the PTFE membrane assay following Janisiewicz et al.^10^, with minor modifications. Briefly, the wells of a 12-well culture plate were filled with 10% PDB (0.6 ml per well) with JBC17WT or the selected mutant strains (1 × 10^8^ cfu/ml). Cylindrical inserts with PTFE membrane (pore size 0.45 μm) attached at the bottom were placed in the wells and allowed to stand for 2 min to ensure the membrane was completely moist. A conidial suspension of *P. digitatum* in distilled water (DW) (0.4 ml of 1 × 10^6^ conidia/ml suspension per cylinder) was then placed in the cylinder, and the plates were incubated at 25 °C. Wells containing only 10% PDB with a DW-filled PTFE cylinder were used as negative controls, whereas wells with 10% PDB and cylindrical inserts with conidial suspension served as positive controls. After 24 h of incubation, the cylinders were removed from the wells, and excess liquid from the membrane was blotted with tissue paper. The membrane was cut out with a clean scalpel and transferred to a glass slide to observe the germination of conidia under a microscope, as described above. A similar set of culture plates was prepared to determine conidial viability after 24 h of interaction with each bacterial strain. In this set, the cylinders were removed from the plate, blotted with sterile tissue paper to absorb excess liquid from the membrane, and placed in another 12-well plate with fresh 10% PDB. The plates were incubated for an additional 24 h at 25 °C, and conidial germination was observed under a microscope.

### Growth assay in nutrient-rich and -poor conditions

Tn mutants were assayed for changes in growth rate under nutrient-limited (10% PDB) and nutrient-rich (100% PDB) conditions. The wild type and selected mutants were cultured overnight in LB at 28 °C and 180 rpm in the dark. The pre-cultures were diluted to the same concentration at OD_600_ and inoculated into 3 ml of fresh nutrient-rich and -poor PDB media (OD_600_ 0.05). The tubes were incubated at 28 °C and 180 rpm, and the growth of each strain was spectrophotometrically monitored by measuring OD_600_ after 12 h and 24 h of incubation for 100% PDB and 10% PDB, respectively.

### Analysis of carbon utilization pattern using Biolog plates and AWCD analysis

The differences in substrate utilization between the wild type and Tn mutants (Tn04-B10 and Tn04-H1) were assayed using a GN2 microplate (Biolog, Inc., Haywood, CA, USA), which has different carbon sources, amino acids, carboxylic acids, amines, alcohols, esters, and others in a 96-well plate. Each bacterial strain was streaked on LB agar, and the colonies were swabbed to make a suspension of 0.2 OD_600_ in GN/GP inoculating fluid (Biolog). A 100 µl suspension was applied to each carbon substrate-containing wells, and the plates were incubated at 28 °C. Plates were monitored for color development, and the absorbance was measured at 595 nm using a microplate reader after 6 h, 16 h, 24 h, and 48 h. Biolog microplate data were normalized by AWCD: AWCD = [Σ(*C* − *R*)]/*n*, where *C* is the absorbance (OD) of each well containing the carbon substrate, *R* is the OD of the no-substrate control well of each plate, and *n* is the number of substrates (*n* = 95). The experiment was performed in triplicate and repeated twice.

### Swarming and swimming motility assay

Motility was assayed using soft-agar plates with 0.5% agar and 0.3% agar for swarming and swimming movements, respectively. To compare motility under various nutrient concentrations, swarming and swimming motility was assayed in nutrient-limited (10% PDA) and nutrient-rich (100% PDA) conditions. Sterilized media were poured onto Petri plates 1 d before the experiment and kept for 1 h in a laminar flow hood. On the following day, plates were air-dried again for 1 h. Each mutant was grown in LB medium with tetracycline (25 μg/ml), and the cell culture (5 μl of OD 0.2) was inoculated into the center of the swarm or swim agar plate^56^. The wild-type strain was inoculated as a positive control. The swarming and swimming movements were measured after incubation at 28 °C for 24 h for 100% PDA and 48 h for 10% PDA, respectively. Experiments were performed in triplicates and repeated twice.

### Complementation of *fliR* gene mutant

The mutant impaired in *fliR* was complemented by Hung et al.^57^. Briefly, the *fliR* gene, including a 100 bp upstream region, was amplified from genomic DNA of wild-type JBC17 by PCR using primers fliRFP and fliRRP (See Supplementary Table S5 online). The chloramphenicol acetyl transferase (CAT) gene from the pKD3 plasmid was amplified using primers cat-sac1FP and cat-sac1RP and cloned into the pUCP18 vector digested with *Sac*I. The derivative (pUCP18*Cat*) was used for this study. The PCR amplicon was gel-purified using the GeneAll Expin gel SV kit, cloned into the pGEM-T easy vector, and then sub-cloned into pUCP18*Cat* at *Hin*dIII/*Bam*HI sites. The plasmids were transformed into competent cells of the mutant Tn04-H1 (*fliR*) by electroporation. The complemented strain (Tn04-H1 + fliR) was confirmed by restriction digestion, and PCR using the primer sets fliRFP and fliRRP. The growth rate, swimming motility, and ability to inhibit conidial germination of the complemented strain were assayed in nutrient-rich and nutrient-limited media and compared to the wild type.

## Supplementary Information

Supplementary Information 1.

Supplementary Information 2.
