# Supplementary material for: High-throughput identification of genes influencing the competitive ability to obtain nutrients and performance of biocontrol in Pseudomonas putida JBC17
Source: Sci Rep. 2022 Jan 18;12:872. doi: 10.1038/s41598-022-04858-z (PMC8766522; doi:10.1038/s41598-022-04858-z)
Supplement: Supplementary file 1 — Supplementary Information 1. [file 41598_2022_4858_MOESM1_ESM.docx]

**Supplementary Data**

**Supplementary Table S1**. Genes identified from Tn mutants of *Pseudomonas putida* JBC17 that showed changes in inhibition potential of conidia germination

| Tn mutant | Inhibition activity^a^ | Biocontrol activity^b^ | Gene name | Expected function or product |
| --- | --- | --- | --- | --- |
| Tn01-F4 | --- | - | *faa1* | AMP-binding acetyl-CoA synthetase |
| Tn01-H6 | --- | -- | *ppx* | exopolyphosphatase |
| Tn02-D3 | --- | - |  | hypothetical protein |
| Tn02-F8 | -- | = |  | Intergenic region (no promoter in the region) |
| Tn04-B10 | + | + | *fliH* | flagellar assembly protein FliH |
| Tn04-H1 | + | + | *fliR* | flagellar type III secretion system protein FliR |
| Tn05-B7 | - | = | *cysM* | cysteine synthase CysM |
| Tn08-B7 | -- | - |  | Hypothetical Protein |
| Tn08-F8 | - | = | *yrbB* | STAS domain-containing protein |
| Tn09-B3 | + | + | *ytcJ* | [amidohydrolase](https://www.ncbi.nlm.nih.gov/nucleotide/CP029693.1?report=gbwithparts&from=6391055&to=6392893&RID=WP7HZWNE015) |
| Tn09-D3 | - | = | *aroA* | 3-phosphoshikimate 1-carboxyvinyltransferase |
| Tn11-D10 | --- | - | *cirA* | TonB-dependent receptor |
| Tn13-C2 | -- | = | *ycII* | YciI family protein |
| Tn15-F5 | -- | - | *dgdA-3* | aminotransferase class III-fold pyridoxal phosphate-dependent enzyme |
| Tn15-H2 | -- | -- | *prop-7* | proline/betaine transporter |
| Tn17-C8 | --- | -- | *lppL* | [Lipopeptide precursor](https://www.ncbi.nlm.nih.gov/nucleotide/CP025738.1?report=gbwithparts&from=281934&to=283328&RID=KJKN9X2E014) |
| Tn18-C1 | --- | = | *pepP* | Xaa-Pro aminopeptidase |
| Tn18-D10 | -- | - | *argG* | Argininosuccinate synthase |
| Tn19-E7 | -- | -- | *serA* | [D-3-phosphoglycerate dehydrogenase](https://www.ncbi.nlm.nih.gov/nucleotide/CP025542.1?report=gbwithparts&from=5232460&to=5234751&RID=KJKTNV6P014) |
| Tn20-B11 | -- | = | *ctpA* | tail-specific protease |
| Tn22-H2 | -- | = |  | integrase catalytic (helix-turn-helix (HTH) domain-containing protein) |
| Tn23-A5 | + | + | *fliA* | RNA polymerase sigma factor FliA |
| Tn25-B1 | -- | - | *csuE* | Spore Coat Protein U domain protein |
| Tn25-H10 | --- | - | *cirA* | putative TonB-dependent receptor BfrD precursor |
| Tn27-F8 | -- | - | *ilvC* | ketol-acid reductoisomerase |
| Tn30-A7 | + | + | *flgG* | [Flagellar basal-body rod protein](https://www.ncbi.nlm.nih.gov/nucleotide/CP025542.1?report=gbwithparts&from=3092361&to=3092855&RID=KT0S265R014) |
| Tn31-A7 | -- | -- | *gltA* | Citrate (Si)-synthase |
| Tn32-D5 | -- | -- |  | DUF934 domain-containing protein |
| Tn34-A12 | -- | = | *ltaE-2* | Low specificity L-threonine aldolase |
| Tn38-F1 | -- | - | *puuE* | Allantoinase PuuE |
| Tn39-E4 | --- | --- | *dnaj* | [Chaperone protein DnaJ](https://www.ncbi.nlm.nih.gov/nucleotide/CP025542.1?report=gbwithparts&from=5186727&to=5187104&RID=KT17JKFN014) |
| Tn40-A6 | -- | = | *-* | Acetyl-CoA C-acetyltransferase |
| Tn41-A9 | -- | - | *folE_2* | GTP cyclohydrolase 1 |
| Tn41-F7 | -- | - | *cysK* | Cysteine synthase A |
| Tn43-A12 | + | + | *fliR* | Flagellar type III secretion system protein FliR |
| Tn44-B4 | + | + | *yaiI* | YaiI/YqxD family protein |
| Tn44-G12 | + | + |  | - |
| Tn46-A9 | --- | - | *hipB* | helix-turn-helix protein |
| Tn46-C6 | -- | - | *lolA* | DUF1329 domain-containing protein |
| Tn48-G11 | -- | = | *lon* | endopeptidase La |
| Tn50-A7 | --- | - | *panB* | 3-methyl-2-oxobutanoate hydroxymethyltransferase |
| Tn50-G7 | + | + |  | - |
| Tn54-F11 | --- | - | *argH* | Argininosuccinate lyase 1 |
| Tn55-F5 | -- | = |  | - |
| Tn55-H2 | -- | = | *betA_4* | choline dehydrogenase |
| Tn58-H2 | -- | -- |  | - |
| Tn59-H7 | -- | - |  | Intergenic region (no promoter in the region) |
| Tn60-B11 | -- | -- | *yecC* | L-cystine ABC transporter ATP-binding protein YecC |
| Tn61-A7 | -- | = | *ctaD* | cytochrome c oxidase subunit I |
| Tn63-C2 | -- | = |  | Intergenic region (no promoter in the region) |
| Tn63-H1 | -- | - | *ilvC* | ketol-acid reductoisomerase |
| Tn64-B7 | -- | = |  | - |
| Tn66-C6 | - | = | *tctB* | tripartite tricarboxylate transporter TctB family protein |

^a^Conidia germination inhibition activity: + denotes increased inhibition; ---, >6-fold decrease; --, <6.0-≥4.0 old decrease; -, <4.0-≥2.0-fold decrease in germination inhibition compared to wild type.

^b^Biocontrol efficacy: +, denotes efficacy more than 20% increase compared to wild type; -, 20%-40% decrease; --, 50% -75% decrease; ---, more than 90% decrease; =, similar biocontrol efficacy to wild type

Supplementary **Table S3**. Classification of Tn inserted genes based on the primary function in the cell

| Primary function | No. of mutants | Frequency |
| --- | --- | --- |
| Amino acid transport and metabolism | 13 | 24.5% |
| Carbohydrate transport and metabolism | 2 | 3.8% |
| Cell motility | 4 | 7.5% |
| Cell wall/membrane/envelope biogenesis | 1 | 1.9% |
| Coenzyme transport and metabolism | 2 | 3.8% |
| Energy production and conversion | 2 | 3.8% |
| General function prediction only | 2 | 3.8% |
| Hypothetical protein | 2 | 3.8% |
| Inorganic ion transport and metabolism | 3 | 5.7% |
| Lipid transport and metabolism | 2 | 3.8% |
| Post-translational modification, protein turnover, and chaperones | 2 | 3.8% |
| Replication, recombination and repair | 1 | 1.9% |
| Transcription, RNA processing, and degradation | 3 | 5.7% |
| Secondary metabolites biosynthesis, transport, and catabolism | 2 | 3.8% |
| Function unknown | 5 | 9.4% |
| Unidentified | 8 | 15.1% |
| Total | 53 | 100% |

*Functional categories based on EggNOG database (http://eggnog.embl.de.) and COG (<http://www.ncbi.nlm.nih.gov/COG/>)

**Supplementary Table S4.** Strains and plasmids used in this study

| Designation | Relevant characteristics | Reference or Sources |
| --- | --- | --- |
| Strains |  |  |
| *Escherchia coli* SM10pir | *thi-1, thr, leu, tonA, lacy, supE, recA::RP4-2-Tc::Mu, λpir, Km^r^* | Jacobs et al., 2003 |
| *E. coli* MGN-617 | *thithr leu tonAlacYglnVsupE ΔasdA4 recA∷RP4 2-Tc∷Mu(pir)*; Km^r^ | Dozois et al., 2000 |
| *Pseudomonas putida* JBC17 | Wild-type; Amp^r^, Tmp^r^, Crb^r^ | Yu and Lee, 2015 |
| Plasmids |  |  |
| pIT2 | IS*lacZ*/hah, Tet^r^, transposon mutagenesis vector | Jacobs et al., 2003 |
| pUCP18 | *Escherichia-Pseudomonas* shuttle vector; Amp^r^ | Schweizer, 1991 |
| pUCP18*cat* | *cat* gene cloned into pUCP18; Amp^r^, Cat^r^ | This study |
| pGem-T easy | TA cloning vector; Amp^r^ | Promega |

*Km^r^, Tet^r^, Amp^r^, Cat^r^, Tmp^r^ and Crb^r^ indicate resistance to kanamycin, tetracycline, ampicillin, chloramphenicol, trimethoprim and carbenicillin, respectively.

**Supplementary Table S5.** Primers used in this study

| Name | Primer Sequence (5’→3’) | Source |
| --- | --- | --- |
| LacZ-211 | TGCGGGCCTCTTCGCTATTA | Jacobs et al., 2003 |
| LacZ-148 | GGGTAACGCCAGGGTTTTCC | “ |
| CEKG-2A | GGCCACGCGTCGACTAGTACNNNNNNNNNNAGAG | “ |
| CEKG-2B | GGCCACGCGTCGACTAGTACNNNNNNNNNNACGCC | “ |
| CEKG-2C | GGCCACGCGTCGACTAGTACNNNNNNNNNNGATAT | “ |
| CEKG-4 | GGCCACGCGTCGACTAGTAC | “ |
|  |  |  |
| fliRFP | GCACTCAAGCTTGTGGCTGGTACAGACTTTCA | This study |
| fliRRP | GCACTCGGATCCGGTCTTCTGTTTTGTCTTCGC | “ |
| cat-sac1FP | GCACTCGAGCTCGAATATCCTCCTTAGTTCCTATTCC | “ |
| cat-sac1RP | GCACTCGAGCTCACGTCTTGAGCGATTGTGTA | “ |


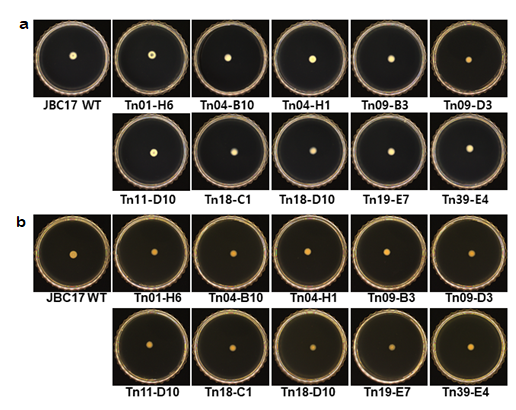


**Supplementary Figure S1**. Swarming motility of *Pseudomonas putida* JBC17 and its Tn mutants. Each strain was grown overnight in LB broth and inoculated in the center of nutrient-poor 10% PDA (A) and nutrient-rich 100% PDA (B) containing 0.5% agar and incubated for 48 and 24 h at 28°C, respectively.
